# Insights about use of p57 in differentiation between complete and partial hydatidiform mole: a scoping review

**DOI:** 10.61622/rbgo/2026rbgo16

**Published:** 2026-05-29

**Authors:** Isabelle Sasso Teixeira, Sheldon Rodrigo Botogoski, Renato Nisihara

**Affiliations:** 1 Universidade Federal do Paraná Paraná Brazil Universidade Federal do Paraná, Curitiba, Paraná, Brazil.

**Keywords:** Hydatidiform mole, Gestational trophoblastic disease, P57 protein, Cyclin dependent kinase inhibitor P57, Immunohistochemistry

## Abstract

**Objective:**

To assess the utility of p57 in differentiating histological subtypes of hydatidiform mole and its applicability in clinical practice.

**Methods:**

A scoping review was conducted following the Joanna Briggs Institute methodology and PRISMA-ScR guidelines. The article search was conducted in the PubMed, EMBASE, BVS, Cochrane, Web of Science, and Scopus databases. Studies published between 2015 and 2025 that evaluated the use of p57 in suspected cases of hydatidiform mole were included.

**Results:**

Nine studies met the inclusion criteria. Most demonstrated high specificity of p57 for CHM diagnosis and a significant reduction in diagnostic discordance compared to morphology alone. However, p57 does not distinguish PHM from non-molar abortions. In addition, rare cases of divergent p57 expression were reported, associated with genetic alterations that may require complementary evaluation using molecular techniques.

**Conclusion:**

IHC for p57 is a widely accessible, low-cost, and effective tool in clinical practice, particularly useful in resource-limited settings. Its routine use, in association with morphology and molecular methods when available, contributes to more accurate diagnoses and safer clinical management of GTDs.

## Introduction

Hydatidiform mole (HM) is an abnormal product of conception with the potential for malignant transformation into gestational trophoblastic neoplasia (GTN). Its incidence is estimated at 1 to 2 per 1,000 pregnancies worldwide, and it can be even more frequent in some regions of the world.^(1,2)^ Gestational trophoblastic disease can be subclassified into complete hydatidiform mole (CHM) and partial hydatidiform mole (PHM). Complete moles typically have an androgenetic diploid genome entirely derived from paternal DNA, while partial moles have a diandric triploid genome, continuing both maternal and duplicated paternal inheritance genetic material.^(3)^ Differentiating between these two histological types is crucial, as CHM presents a higher risk of progressing into GTN – occurring in about 20% of cases – opposed to 5% in PHM.^(4)^

Post-molar follow-up is typically performed with weekly beta-hCG measurements (used as a tumor marker for the disease) until the results are negative, followed by monthly testing. In CHM cases, a six-month surveillance after a negative result is required before monitoring can be safely discontinued. Conversely, in PHM cases, post-molar GTN rarely occurs after beta-hCG normalization, requiring only one month re-evaluation after negative test.^(4,5)^ When the histological subtype cannot be safely determined, follow-up is conducted as in CHM, leading to higher costs to the healthcare system. In the other hand, HM is frequently erroneously classified as abortion resulting in diagnostic delay, which can potentially impact prognosis leading to poorer outcomes in cases of malignancy.^(6)^

The differentiation between the two histological types can be challenging, as both histological types and other products of conception, such as abortions, can share similar histopathological characteristics.^(7)^ This overlap contributes to significant diagnostic variability among different observers, found even in experienced pathologists.^(7,8)^ Genotyping is considered the gold standard method for diagnosis, as it enables the assessment of both ploidy and genetic origin. However, its clinical use is still limited due to high cost and low availability.^(3,9)^ In this context, the use of immunohistochemistry for evaluating the cyclin-dependent kinase inhibitor p57 is helpful in differentiating the histological types of HM.^(10)^ p57 is a protein encoded by the CDKN1C gene, located on chromosome 11, with exclusive maternal allele expression and paternal allele imprinting (silenced). Thus, CHM does not express p57, since exclusive paternal genetic material is present; while expression is found in PHM.^(3,11)^ This difference in expression makes p57 detection a valuable diagnostic tool, especially in laboratories with limited access to molecular techniques. However, discordant cases between the expected pattern of p57 expression and the final diagnosis have been reported, either due to the presence of mutations, mosaicism, or technical limitations.^(10,12)^ Additionally, expression of p57 sensitivity and specificity as a diagnostic tool vary among studies.^(7,13)^

The objective of this scoping review is to evaluate the utility of p57 in differentiating the histological types of HM and its applicability in clinical practice. Secondary objectives include comparing the use of p57 with other methods and defining its advantages and limitations.

## Methods

This study was conducted following the Joanna Briggs Institute methodology for scoping reviews.^(14)^ The following question was formulated: "What is the utility of p57 in differentiating the histological types of hydatidiform mole?". Based on this question, the search was guided by the "Population, Concept and Context" strategy, structured as follows: Population – women diagnosed with HM; Concept – evaluation of p57 protein expression as a diagnostic tool in differentiating between histological subtypes of HM; Context – contribution of the p57 test to clinical management.

This study followed the PRISMA-ScR guidelines, in accordance with the EQUATOR network.^(15)^

### Search strategy

The article search was conducted in the PubMed, EMBASE, BVS, Cochrane, Web of Science, and Scopus databases in June 2025. The descriptors used were "gestational trophoblastic disease", "hydatidiform mole", "molar pregnancy", "p57 protein", "p57kip2", "cyclin-dependent kinase inhibitor p57", combined with the aid of Boolean operators "AND" and "OR". A temporal filter was applied to select articles published in the last 10 years (from 2015 to 2025). (Supplementary Materials - Chart 1S).

### Selection process and analysis

Two independent researchers evaluated the titles and abstracts for article selection. Any disagreements were resolved by a third researcher. Original studies addressing the role of p57 in the differential diagnosis between CHM and PHM and its applicability in clinical practice were included. Case reports, literature reviews, qualitative studies, animal model research, or studies in foreign languages other than English or Portuguese were excluded. Studies that did not directly evaluate p57 expression or its application in the context of gestational trophoblastic disease were also excluded.

### Quality assessment

The studies chosen for full-text reading had their methodology evaluated using the Quality Assessment of Diagnostic Accuracy Studies (QUADAS-2) tool.^(16)^ The study was registered on the OSF platform with registration https://doi.org/10.17605/OSF.IO/4BW7G.

## Results

**Figure 1:**
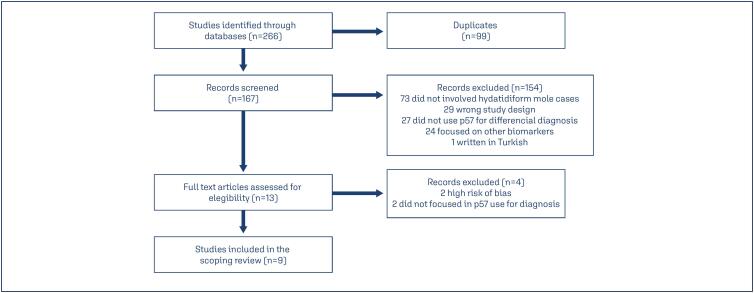
Flowchart of the systematic search and article selection process

Initially, 266 articles were identified, as per the flowchart (Figure 1). After applying the criteria, 13 selected studies were read in full. Of these, two were excluded because they only mentioned the use of p57 with an objective other than method evaluation, and another two were excluded due to a high risk of methodological bias after evaluation with QUADAS-2. Ultimately, nine articles were eligible for this scoping review.

Of the nine articles, seven were retrospective cohort studies, one was a prospective study, and one was a cross-sectional study. Molecular methods were employed in five studies, with two utilizing fluorescence in situ hybridization (FISH) and two using Short Tandem Repeats (STR) genotyping in all cases. One of them used molecular methods only in inconclusive cases. In the study by Usui et al.,^(17)^ immunohistochemistry was evaluated in conjunction with FISH. The main findings of each article are summarized in chart 1. The QUADAS-2 assessment of the included studies is presented on Supplementary Materials (Figure 1S).

## Discussion

In this review, a consensus was observed among the included authors that the isolated use of the morphological method with hematoxilin and eosin presents high rates of diagnostic error. Discordance between morphological diagnosis and that performed by immunohistochemistry and genotyping was observed in 9% of cases in Triratanachat et al.'s^(19)^ study, 26% in López et al.'s^(21)^ study, and an alarming 33% in Zainal et al.'s^(24)^ study, proving the necessity of using ancillary techniques in the evaluation of suspected HM cases.^(19,21,24)^ Furthermore, performing immunohistochemistry with identification of the p57 protein is associated with better accuracy in the differential diagnosis of HM.^(26)^ Immunohistochemistry for p57 is often the first complementary technique used in cases of diagnostic question due to its easy accessibility.^(27)^ However, most gestational trophoblastic disease guidelines – both international and Brazilian – do not include its routine use in pathological diagnosis in cases of gestational loss or suspected HM.^(1,5,28)^

Most studies have shown the high accuracy of p57 for diagnosing CHM cases,^(18,20,22,23,25)^ with a negative p57 immunohistochemistry result being highly predictive of this type of mole. Such findings underscore the importance of routine p57 immunohistochemistry use, highlighting that complete moles have a higher risk of malignant evolution and poorer outcomes, and non-diagnosis can lead to delays in initiating appropriate treatment.^(6,24,25)^ On the other hand, cases of over-diagnosis of CHM are also detrimental, as they unnecessarily increase follow-up time and costs for the healthcare service; in addition to impacting patients’ emotional aspects, increasing anxiety and depression rates associated with diagnosis.^(24,29)^

Beyond its diagnostic importance, some authors demonstrated the utility of p57 as a prognostic marker, showing that cases presenting p57 negativity were associated with a higher rate of persistent disease and progression to GTN.^(19)^ Such a correlation can be attributed to p57's function as a cyclin-dependent cell cycle inhibitor, acting in the G1 to S phase transition.^(30)^ Thus, its absence, characteristic of androgenetic complete moles, results in the loss of cell proliferation control, favoring a more aggressive biological behavior and higher potential for malignancy.^(31)^ Similarly, Erol et al.^(18)^ reported that p57 acts as a tumor suppressor and its absence, combined with overexpression of epidermal growth factor receptor type 2 (c-erbB-2) and alterations in Bcl-2 and CD117 expression, was associated with higher proliferative potential and risk of progression to persistent disease. López et al.^(21)^ also identified progression to invasive mole in a case with p57 absence and c-erbB-2 amplification, reinforcing the hypothesis that alterations in cell proliferation control are associated with worse prognosis. Xing et al.^(23)^ observed that heterozygous CHM have a greater propensity for progression to GTN than homozygous ones, attributing this behavior to the duplicated genetic load and increased trophoblastic proliferative activity.

**Chart 1:** Synthesis of the studies included in the review

Author/Year	Country/Sample size	Purpose/IHC p57	Findings	Main conclusion
Erol et al. (2016)^(18)^	Turkey n=67	To assess the use of p57, HER2, CD117, and Bcl-2 in distinguishing complete hydatidiform mole (CHM), partial hydatidiform mole (PHM), and hydropic abortion (HA). Used mouse monoclonal anti-p57 (clone 25B2; 1:50)	All CHMs were p57-negative; PHMs and HAs showed similar p57 positivity (74%). Genotyping used in 7 equivocal cases: confirmed 4 CHMs, 2 PHMs, 1 HA.	p57 immunohistochemistry (IHC) is highly specific for CHM but not useful for distinguishing PHM from HA. Combining p57 with other markers (like HER2, CD117, Bcl-2) and genotyping improves diagnostic accuracy in challenging cases.
Triratanachat et al. (2016)^(19)^	Thailand n=127	To evaluate the utility of p57 IHC in distinguishing CHM and PHM and identify key morphological features. Used mouse monoclonal anti-p57 (clone KP39, 1:200 Neomarker®)	Concordance between hematoxilin and eosin (H&E) and p57 was 90.6%; 12 cases (9.4%) had discordant diagnosis. 107 CHM (p57-negative) and 20 PHM (p57-positive) after IHC. 34/107 p57-negative cases developed postmolar GTN versus. 1/20 p57-positive cases.	p57 IHC improves diagnostic accuracy and should be used in all suspected cases of hydatidiform mole (HM). Histopathology alone has limitations, especially in early gestation. Molecular studies may be required in equivocal or discordant cases.
Samadder et al. (2017)^(20)^	India n=53	To evaluate the role of p57 IHC in combination with histomorphology for differentiating between CHM, PHM, and HA. Used p57Kip2 Ab-6 antibody (Thermo Fisher Scientific®).	96% of CHMs were p57-negative; 100% of PHMs and 95% of non-molar controls were p57-positive. 4 of 27 molar cases (14.8%) were discordant between initial morphology and IHC. 2 of 13 HA cases were reclassified as molar after IHC. One CHM case showed inconsistent staining, possible biparental mole or maternal chromossome (chr) 11 retention.	p57 is reliable to identify CHM and is a valuable adjunct to morphology, especially in early gestation and ambiguous cases. Cannot reliably distinguish PM from HA, but aids significantly in improving diagnostic accuracy.
López et al. (2020)^(21)^	Brazil n=108	To evaluate the utility of p57 IHC and HER2 Fluorescence In Situ Hybridization (FISH) using tissue microarray in the classification of HM. Used mouse monoclonal anti-p57 (clone Kp10/SP118, DAKO®); combined with HER2 FISH ploidy analysis.	H&E: 57 CHM, 47 PHM, 4 inconclusive p57: 55% negative, 20% positive, 25% inconclusive HER2 FISH: 63% diploid, 30% triploid, 5% inconclusive Diagnostic changes occurred in 28 cases (26%), mainly from PHM to CHM. 73% of diagnoses were supported by p57; 71% by FISH; 75% by morphology	The combination of histopathology, p57 IHC and HER2 FISH improves diagnostic accuracy in molar pregnancies. They have analyzed the use of p57 in association with the FISH HER2 technique, but they highlighted that p57 alone was responsible for 50% of diagnostic changes in the study.
Awosusi et al. (2020)^(22)^	Nigeria n=100	To assess the value of p57 IHC as an ancillary tool for differentiating CHM and PHM. Used rabbit monoclonal antibody (clone EP2515Y, Abcam®).	Original H&E diagnosis: 57 CHM, 37 PHM, 6 equivocal HMs; After p57 IHC: 68 CHM, 32 PHM. 10 cases reclassified: 8 initially PHM → CHM, 2 initially CHM → PHM 5/6 equivocal HMs reclassified as CHM No discordant staining patterns observed.	p57 IHC is a reliable marker for distinguishing CHM from PHM and improves diagnostic accuracy, particularly in morphologically ambiguous or equivocal cases. Recommended as routine adjunct in histopathological diagnosis of HMs.
Xing et al. (2021)^(23)^	EUA n=2217	Prospective cohort to refine the diagnosis of HM using an algorithm incorporating p57 IHC and STR genotyping. Used mouse monoclonal anti-p57 (prediluted, Neomarkers®).	99.8% of CHMs were p57-negative and 96.7% androgenetic 99% of PHMs were p57-positive and 97% diandric triploid 56 mosaic cases: 37 with p57-negative CHM component 5 CHMs were biparental (likely familial); 3 PHMs were p57-negative due to loss of maternal chr11 H&E alone had 55–75% accuracy; p57 + genotyping improved classification to 94%	p57 is highly reliable for CHM diagnosis and correlates strongly with genotyping. However, interpretation must be integrated with morphology and genotyping to address unusual presentations (e.g., mosaicism, familial CHMs, donor egg gestations).
Zainal et al. (2021)^(24)^	Malaysia n=82	To determine the role of p57 IHC in improving diagnostic accuracy of HM subtypes in comparison to initial H&E evaluation. Used mouse monoclonal anti-p57 Ab-6 clone 57P06 (Thermo Scientific, RTU®).	H&E initially classified 39 CHM, 41 PHM, 2 unclassified. After p57 IHC, 66 CHM, 14 PHM, 2 non-molar. 33% diagnostic discrepancy between H&E and IHC results. 27 PHMs reclassified as CHM after IHC.	p57 IHC significantly improves diagnosis of HM subtypes and should be routinely used in conjunction with H&E, especially in resource-limited settings. Limitations include inability to distinguish CHM from HA.
Wong et al. (2021)^(25)^	Malaysia n=51	To assess the combined diagnostic accuracy of p57 IHC and DNA ploidy analysis by FISH in differentiating CHM, PHM, and non-molar abortion (NMA). Used rabbit monoclonal anti-p57 (Abcam®); DNA FISH for chr 11/16 and X/Y.	H&E diagnosis: 18 CHM, 24 PHM, 9 NMA Final reclassification: 27 CHM (p57-/diploid), 9 PHM (p57+/triploid), 15 NMA (p57+/diploid) Diagnostic accuracy of H&E alone: CHM 78.4%, PHM 70.6%, NMA 88.2% 2 p57-discordant cases were excluded.	H&E alone lack accuracy in differential diagnosis between HM types and NMA. p57 IHC is highly specific for CHM but insufficient alone to distinguish PHM from NMA. Combined with DNA ploidy analysis, it enhances diagnostic precision. An algorithmic approach is recommended in routine practice.
Usui et al. (2024)^(17)^	Japan n=80	To evaluate the diagnostic value of combining p55 IHC and FISH analysis compared to STR genotyping for differentiating HM from NMA. Used rabbit polyclonal anti-p57 (Ab-7, clone RB-1637-R7, Thermo Fisher®)	44 CHMs (p57- / diploid), 20 PHMs (p57+ / triploid), 14 non-molar abortions (p57+ / diploid) 10 cases had initial misdiagnoses corrected after FISH (8 PHMs misclassified as abortion; 2 abortions as PHM) 2 mosaic cases showed discordant p57 staining (cytotrophoblast +, stroma −) 1 CHM was p57-positive due to retained maternal chr11 (false negative)	Combined use of p57 IHC and FISH increases diagnostic accuracy, particularly in ambiguous or discordant cases and distinguishing PHM from abortion. Some rare exceptions, like mosaicism and maternal chromosome retention, highlight the need for integrated diagnostic workflows.

Although Usui et al.'s study^(17)^ evaluate the use of p57 in combination with the FISH technique, it was included in this review for significantly contributing to the understanding of p57's diagnostic limitations, particularly in distinguishing between PHM and hydropic abortion, conditions that share positivity for this marker. However, similar to what was observed in other studies, the authors also reported high specificity of p57 in identifying CHM cases.^(17)^ Generally, articles widely recommend performing p57 immunohistochemistry as a screening method in all suspected cases of molar pregnancy, reserving molecular analyses for cases with positive p57 expression where diagnostic doubt between PHM and hydropic abortion persists.^(19,22,24)^

Rare cases of divergent or unexpected p57 expression were identified. Samadder et al. ^(20)^ described a case of CHM with positive p57 expression, raising the hypothesis of maternal chromosome 11 retention in an androgenetic conceptus – a rare phenomenon in which residual maternal genetic material is inherited at the time of conception, resulting in positive p57 expression in complete moles.^(25,32)^ This alteration was confirmed in a case described by Wong et al.,^(25)^ who demonstrated 46,XX diploidy associated with trisomy of chromosome 11 using the FISH technique. Cases of chromosome 11 loss were also reported, leading to the occurrence of PHM with negative p57 expression, as described by Xing et al.,^(23)^ who diagnosed the alteration by STR genotyping, revealing triploidy. This condition can generate false-positive diagnoses of CHM and was also suspected in two cases of erroneous p57 expression reported by Erol et al.^(18)^ Additionally, Xing et al.^(23)^ reported five cases of CHM with biparental genotype and negative p57 expression, suggesting the presence of a possible familial syndrome associated with mutations in the NLRP7 gene. López et al.^(21)^ also reported similar cases of CHM with triploidy and negative p57. Such cases are extremely rare, frequently associated with recurrent HM, and their correct diagnosis is only possible through genotyping, highlighting the limitations of isolated p57 immunohistochemistry use.^(21,23)^

**Figure 2:**
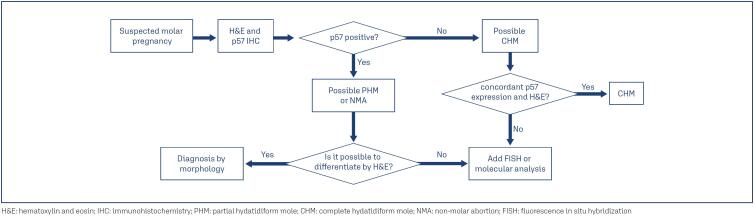
Flowchart gestational trophoblastic disease differential diagnosis

The integration between morphology, p57 immunohistochemistry, and molecular methods allows for the construction of more precise diagnostic algorithms, as suggested by some authors, and should be implemented whenever possible.^(19,23,25)^ Through the study of the included articles, we suggest a flowchart (Figure 2) for the differential diagnosis of the different histological subtypes, as a way to optimize resources and improve the diagnosis of gestational trophoblastic diseases.

It is worth noting that genotyping and more refined molecular methods may not yet be a reality in many places, especially in developing countries. On the other hand, the use of p57 immunohistochemistry in the routine of suspected molar pregnancy cases can be considered a more accessible, relatively low-cost, and highly reliable method for clinical practice, being much more accurate than the isolated use of the morphological method.^(17,22)^

## Conclusion

The use of the p57 marker via immunohistochemistry enhances the diagnostic accuracy of HM, particularly in distinguishing between CHM and PHM or hydropic abortion. Although it presents limitations in rare situations, its use combined with morphology and, when necessary, molecular methods, contributes to more precise diagnoses and safer clinical management, being routinely recommended in suspected cases of molar pregnancy.

## Data Availability

The research data are described in the article presented.
