# Supplementary material for: Insights about use of p57 in differentiation between complete and partial hydatidiform mole: a scoping review
Source: Rev Bras Ginecol Obstet. 2026 May 29;48:e-rbgo16. doi: 10.61622/rbgo/2026rbgo16 (PMC13399369; doi:10.61622/rbgo/2026rbgo16)
Supplement: Supplementary Material [file 1806-9339-rbgo-48-e-rbgo16-Suppl01.pdf]

Supplementary material

Chart 1S. Search strategy

PubMed (90)	("Gestational Trophoblastic Disease"[MeSH Terms] OR "Hydatidiform Mole"[MeSH Terms] OR "Gestational Trophoblastic Disease"[Title/Abstract] OR hydatidiform mole[tiab] OR hydatidiform moles[tiab] OR molar pregnancy[tiab] OR molar pregnancies[tiab] OR "molar pregnancy"[Title/Abstract]) AND ("p57"[Title/Abstract] OR "p57kip2"[Title/Abstract] OR "cyclin-dependent kinase inhibitor p57"[Title/Abstract]) AND (y_10[Filter])
EMBASE (78)	('gestational trophoblastic disease'/exp OR 'hydatidiform mole'/exp OR 'molar pregnancy':ti,ab,kw OR 'gestational trophoblastic disease':ti,ab,kw OR 'hydatidiform mole':ti,ab,kw) AND ('p57 protein':ti,ab,kw OR 'p57kip2':ti,ab,kw OR 'cyclin-dependent kinase inhibitor p57':ti,ab,kw) AND [2015-2025]/py AND 'article'/it
Scopus (54)	TITLE-ABS-KEY ("gestational trophoblastic disease" OR "hydatidiform mole" OR "molar pregnancy") AND TITLE-ABS-KEY ("p57 protein" OR "p57kip2" OR "cyclin dependent kinase inhibitor p57" OR "CDKN1C") AND PUBYEAR > 2014 AND PUBYEAR < 2026 AND (LIMIT-TO (DOCTYPE, "ar")
Web Of Science (23)	TS=("hydatidiform mole" OR "molar pregnancy" OR "gestational trophoblastic disease") AND TS=(p57kip2 OR "p57 protein") Timespan: 2015 to 2025 (Index Date)
BVS (15)	tw:("gestational trophoblastic disease" OR "hydatidiform mole" OR "molar pregnancy") AND tw:(p57 OR p57kip2 OR cdkn1c OR "cyclin dependent kinase inhibitor p57") AND (year_cluster:[2015 TO 2025]) AND instance:"regional"
Cochrane (6)	("gestational trophoblastic disease":ti,ab,kw OR "hydatidiform mole":ti,ab,kw OR "molar pregnancy":ti,ab,kw) AND ("p57":t OR "p57kip2":ti OR "cyclin dependent kinase inhibitor p57":ti) Publication date from 2015 to 2025.

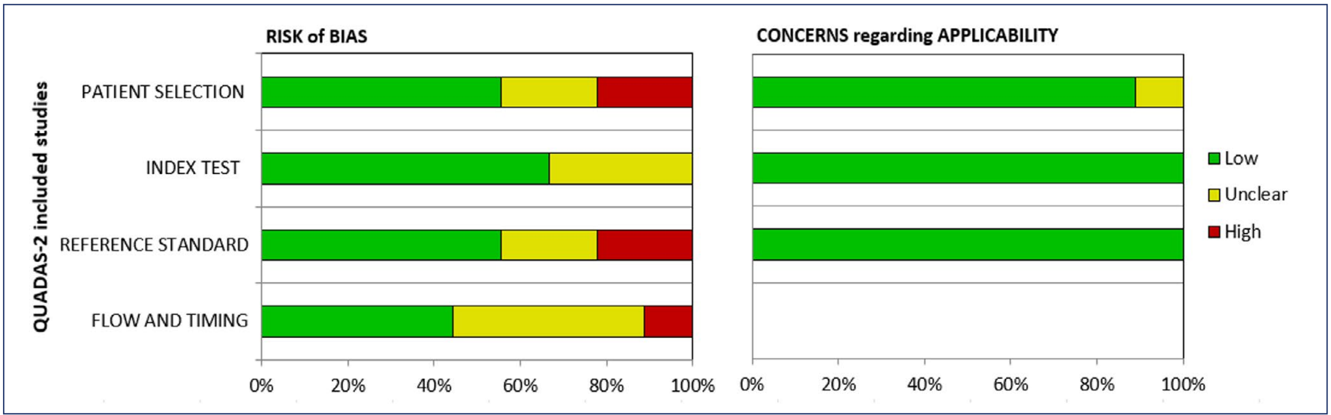

Figure 1S. QUADAS-2 assessment of the included studies
